# The Regenerative Effects of c-Met Agonistic Antibodies in Vocal Fold Atrophy

**DOI:** 10.3390/ijms23147818

**Published:** 2022-07-15

**Authors:** Hyunsu Choi, Seung-Shin Yu, Jiwon Choi, Choung-Soo Kim

**Affiliations:** 1 Clinical Research Institute, Daejeon St. Mary’s Hospital, Daejeon 34943, Korea; 20110201@cmcnu.or.kr; 2 R&D Center for Innovative Medicines, Helixmith Co., Ltd., Seoul 07794, Korea; seungshin@helixmith.com; 3 Department of Otolaryngology-Head and Neck Surgery, College of Medicine, The Catholic University of Korea, Seoul 06591, Korea; cjw412@gmail.com

**Keywords:** vocal fold atrophy, hepatocyte growth factor, c-Met agonistic antibody

## Abstract

Background: Atrophy of the vocal folds and the accompanying glottic insufficiency affect the quality of life. Although growth factors have been used to treat muscle atrophy, their effectiveness is limited by their short half-life. Methods: In total, 15 rabbits and 24 rats were used for the study. The right recurrent laryngeal nerves of all animals were transected. One month following nerve transection, PBS (PBS group), rHGF (HGF group), or a c-Met agonistic antibody (c-Met group) was injected into the paralyzed vocal folds. The larynges of the rabbits were harvested from each group for histologic examination and subjected to PCR analysis. Results: Cross-sectional areas (CSAs) of thyroarytenoid muscles were evaluated. The c-Met group had increased CSAs compared to the PBS and HGF groups, but there were no significant differences compared to normal controls. The expression levels of myogenesis-related genes were evaluated three weeks after the injection. The expression levels of myosin heavy chain IIa were significantly increased in the PBS group, while the expression levels of MyoD were increased in the c-Met group. Conclusions: The c-Met agonistic antibody showed promise for promoting muscle regeneration in a vocal fold palsy model.

## 1. Introduction

Glottic closure is essential for vocalization and airway protection [1]. Several pathological conditions cause glottic insufficiency, such as vocal fold paralysis and presbylarynx, which is the age-related atrophy of laryngeal muscle [2,3,4]. Damage to the recurrent laryngeal nerve (RLN) causes vocal-fold paralysis and intrinsic muscle atrophy [5]. Thyroarytenoid (TA) is the main intrinsic laryngeal muscle involved in glottic closure [6].

Space-occupation strategies, such as laryngeal framework surgery or injection laryngoplasty, have recently been used for the treatment of glottic insufficiency, but could not improve or prevent laryngeal muscle atrophy. Laryngeal reinnervation surgery can prevent intrinsic muscle atrophy in vocal fold paralysis, but is technically difficult, requires neck incisions, and may lead to misdirected regeneration and nerve sacrifice [7,8,9].

Several studies have reported that hepatocyte growth factor (HGF) has anti-fibrotic effects and prevents muscle atrophy. A recent phase II/III clinical trial demonstrated that weekly intracordal HGF injections for four weeks effectively faded scars and improved the presbylarynx [10]. Sheean et al. reported that exogenous HGF stimulated muscle repair, but the effects were limited due to the short half-life (2.4 min) and required repeat injections [11].

c-Met is the transmembrane tyrosine-kinase receptor for HGF. Antibodies that activate c-Met receptors, such as HGF, are more stable and have longer half-lives, and they could potentially overcome the limitations of HGF. In this study, we compared the effects of rHGF and a c-Met receptor agonistic antibody on vocal atrophy in a rabbit vocal fold model.

## 2. Results

### 2.1. Histological Examination

**Figure 1:**
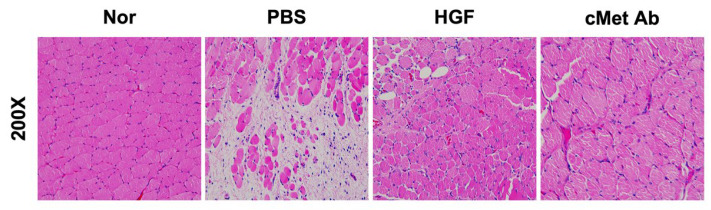
Representative histological images of H&E staining of specimens from the normal, PBS, HGF, and c-Met groups at three weeks post-injury (original magnification: 200×).

A histological examination was performed using a rabbit vocal fold palsy model. The right TA muscles were evaluated three weeks following injection. The TA muscle demonstrated greater atrophy in the PBS group compared to other groups (Figure 1).

**Figure 2:**
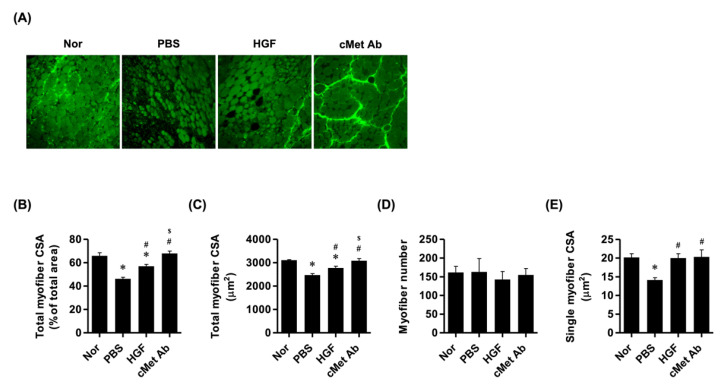
Histological evaluation of laryngeal muscles. (**A**) Representative images of myosin heavy-chain staining (original magnification: 200×). (**B**,**C**) Cross-sectional area of the total muscle fibers. (**D**) The number of muscle fibers was quantified by counting single muscle fibers. (**E**) Cross-sectional area of a single muscle fiber. * *p* < 0.05 compared to Nor (normal); # *p* < 0.05 compared to PBS; $ *p* < 0.05 compared to HGF.

The cross-sectional areas (CSAs) of the TA muscles were analyzed in each group. The PBS group had significantly decreased intrinsic muscle CSA compared to normal controls, while the HGF group had increased CSA compared to the PBS group, although it was still lower than in the control group. The c-Met group had significantly increased CSAs compared to the PBS and HGF groups, with no significant differences compared to controls. There were no differences in myofiber numbers among the groups, but the single-myofiber CSAs were increased in the HGF and c-Met groups compared to the PBS group (Figure 2).

**Figure 3:**
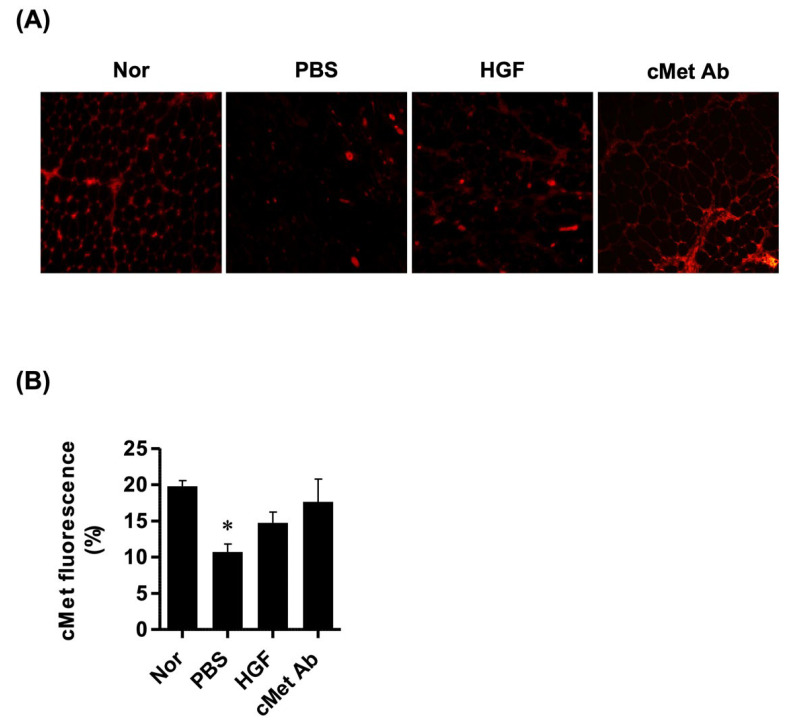
Analysis of c-Met expression in laryngeal muscles. (**A**) Representative images of c-Met staining (original magnification: 200×). (**B**) Relative intensity of c-Met fluorescence. * *p* < 0.05 compared to Nor (normal).

The expression levels of c-Met in TA muscles were evaluated. c-Met expression was significantly decreased in the PBS group compared to normal controls, while it was insignificantly increased in the HGF and c-Met groups (Figure 3).

### 2.2. Gene Expression Analysis

The expression levels of myogenesis-related genes were evaluated three weeks after injection in the rat vocal fold palsy model. The expression level of myosin heavy chain IIa was increased significantly more in the PBS than in the HGF and c-Met groups.

**Figure 4:**
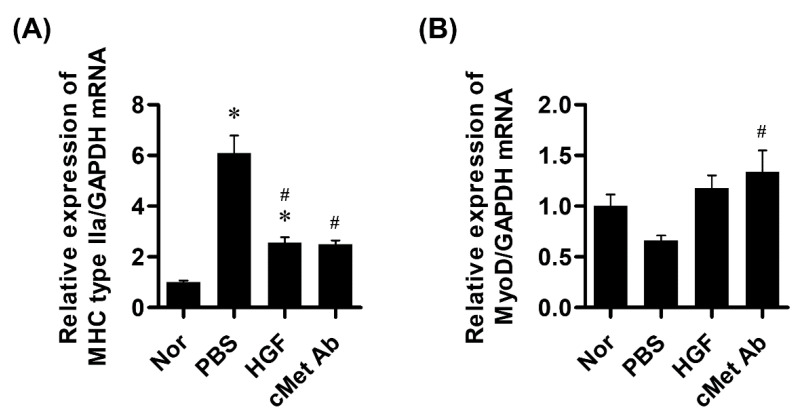
Results of real-time PCR of myogenesis-related genes. (**A**) The expression level of myosin heavy chain IIa. (**B**) The expression level of MyoD. * *p* <0.05 compared to Nor (normal); # *p* <0.05 compared to PBS.

The expression levels of MyoD were decreased in the PBS group compared to the control group, but there was no significant difference. Only the c-Met group showed a significant increase compared to the PBS group (Figure 4).

## 3. Discussion

Growth factors have been extensively studied, including for skeletal muscle growth and regeneration [12]. Several studies have demonstrated the effects of growth factors on vocal-fold regeneration, but only if delivered directly into the tissue. The effects of growth factors might be limited due to their short half-life. Therefore, recent studies have focused on obtaining slow-release substances with the same effects.

Hiwatashi et al. used a collagen-gelatin sponge containing growth factors that were released slowly during degradation [13]. Choi et al. used small-intestinal submucosa gel for controlled release of growth factors in a vocal fold wound-healing animal model [14]. Kwon et al. introduced PCL/F127 for controlled release and vocal fold augmentation in an animal model of vocal fold palsy [15].

However, there are some problems with controlled-release materials. First, most slow-release materials must remain in the tissue for long periods and cause unintended volume-increase effects until their decomposition. Second, there are no substances currently available that have viscoelastic properties similar to vocal folds. Viscoelastic properties are important for vocal-fold vibration. Injected materials affect the viscoelastic properties and interfere with normal vocal fold vibrations. Therefore, it is better to avoid injecting substances into the vocal folds for the purpose of controlled release, unless volume increase is also required.

In this study, we used HGF for vocal fold muscle regeneration in an animal model of vocal fold palsy. HGF is present in the extracellular matrix near satellite cells and is released when injured or stretched. It then binds to the c-Met receptors of quiescent satellite cells and induces their activation [16]. c-Met agonistic antibodies bind preferentially to c-Met receptors and activate them for longer durations. Previous studies have reported that the serum half-life of a c-Met agnostic antibody was approximately three days, and it remained in situ for six days when injected intravenously [17]. However, in this study, c-Met was injected directly into the TA muscles to further increase the effect time. An agonistic antibody could compensate for the short half-life of HGF and may reduce or eliminate the need for controlled-release materials.

In this study, c-Met expression was increased in the c-Met group compared to the PBS group, and the gene expression levels for muscle regeneration were increased two weeks after the injection. These changes resulted in histological differences. Interestingly, CSAs of single and total myofibers were significantly increased, but the number of muscle fibers was not different among the groups. These results indicate that c-Met and HGF cause hypertrophy of the muscle fibers, instead of increasing the number of muscle fibers for the regeneration of vocal folds.

In TA muscles, several types of MHC isoforms and their compositions were affected by the developmental, hormonal, and neuronal status. Previous studies showed that, among the MHC isoforms, the expression of type IIa increased following denervation [18,19,20]. However, MyoD, known to be an important protein for regulating muscle differentiation, was increased during muscle hypertrophy [21]. The increase in MHC type IIa and MyoD expression levels was consistent with previous studies.

There were some limitations to our study. First, histologic examination was performed three weeks after the injection. Although the c-Met agonistic antibody showed better effects than HGF, since it acts for a longer period than HGF, it is expected that the difference will be even greater after 2–3 months. Therefore, further long-term studies are required for validation of our results. Second, the mRNA expression data for myogenesis-related genes were from rat models. It is preferable to perform PCR tests in rabbit models. However, a larger sample size is required to obtain statistical significance. Some studies used rabbits for PCR, but the number of animals was too small to obtain statistical significance. In this study, a total of 24 rat larynges were harvested for PCR. Finally, although changes to the MHC isoform during muscle atrophy have already been studied, identifying changes to the MHC isoform’s composition in laryngeal muscle in each group might help us to better understand the effects of denervation, HGF, and the c-Met agonistic antibody. Further studies are needed on these topics.

In conclusion, injection of a c-Met agonistic antibody into atrophied vocal folds, as caused by vocal fold palsy, regenerated vocal muscles, which we revealed through histological and PCR examinations. Our results showed the promising effects of c-Met agonistic antibodies for muscle regeneration in a vocal fold palsy model.

## 4. Materials and Methods

### 4.1. Protein Expression in Rat Model

The study was approved by the Animal Ethics Committee of the Catholic University of Korea (permit no. CMCDJ-AP-2020-007). The animals were cared for in accordance with established institutional guidelines.

A total of 24 Sprague-Dawley rats were anesthetized via intraperitoneal injections of ketamine hydrochloride (100 mg/kg) and xylazine hydrochloride (10 mg/kg). The animals were placed in a semivertical position on a custom platform. A vertical incision was made at the midline of the neck. The strap muscles were dissected until the thyroid gland and trachea were encountered, and the thyroid isthmus was divided. The right RLN was identified near the inferior thyroid artery on the medial side of the thyroid gland. After right RLN transection, right-sided vocal fold palsy was endoscopically confirmed using a pediatric endoscope.

One month following the nerve transection, the rats were anesthetized again and either 100 ng of rHGF (MilliporeSigma, Burlington, MA, USA) or 100 ng of c-Met-agonistic antibody (c-Met Ab (VM507); Helixmith, Seoul, South Korea) [17], dissolved in 50 μL phosphate-buffered saline (PBS), was injected into the TA muscle in the experimental groups. In the control group, 50 μL of PBS was injected. Injections were performed using a syringe equipped with a 30-gauge long needle, under direct vision, using a pediatric laryngoscope.

The right vocal folds were harvested from all groups two weeks after the injections for polymerase chain reaction (PCR) testing.

### 4.2. Muscle Regeneration in Rabbit Model

Fifteen New Zealand white rabbits (Damul Science, Daejeon, South Korea) weighing 3 kg was used to study the vocal fold atrophy. The animals were divided into three groups for the PBS, rHGF, and C-Met agonistic Ab treatments. After premedication with xylazine (Rompun 10 mg/kg; Bayer, Leverkusen, Germany), the rabbits were anesthetized via intramuscular injection of 50 mg/kg ketamine, and they were placed in dorsal recumbency. The neck area was shaved and a vertical incision was made in the midline of the neck. The strap muscles were dissected until the thyroid gland and trachea were encountered. The thyroid isthmus was divided. The right RLN was identified near the inferior thyroid artery on the medial side of the thyroid gland, and it was transected. Endoscopic examination was then performed in all rabbits.

One month after nerve transection, 100 ng of rHGF in 100 μL PBS (HGF group) or 100 ng of c-Met agonistic Ab in 100 μL PBS (c-Met group) was injected into the paralyzed vocal folds. A syringe equipped with a 25-gauge long needle was used for injections under direct vision employing a 0° endoscope. The correct injection site was confirmed by vocal fold bulging, which reflected the injected volume.

The larynges of rabbits in all groups were harvested for histologic examination three weeks after the injection. Specimens were embedded in paraffin blocks and 4-μm-thick coronal sections were made using a microtome. The specimens were stained with hematoxylin and eosin (H&E) using standard pathology department protocols. The stained samples were observed under a light microscope (Eclipse TE300; Nikon, Tokyo, Japan).

### 4.3. RNA Isolation and Real-Time Reverse Transcription PCR in Rat Vocal Folds

Rat vocal folds were harvested two weeks after injection and homogenized with TissueLyser II (Qiagen, Hilden, Germany). Total RNA was extracted using TRIzol reagent (Invitrogen, Waltham, MA, USA) according to the manufacturer’s instructions. Complementary DNA (cDNA) was synthesized from 1 μg of RNA using a Reverse Transcriptase Premix Kit (Elpis Biotech, Daejeon, South Korea). Real-time PCR was then performed using an ABI 7500 FAST system (Applied Biosystems, Waltham, MA, USA) in a 20-μL reaction mixture containing Power SYBR Green PCR Master Mix (Applied Biosystems), 500 nM forward and reverse primers, and 1 μL cDNA. The gene expression levels were determined by normalizing to GAPDH using the 2^−∆∆CT^ method. The following primers were used: GAPDH: F, ACCACAGTCCATGCCATCAC, R, TCCACCACCCTGTTGCTGTA; MHC type IIa: F, TTGCTCTACCCAACCCTAAGGATG, R, TTGTGTTTCTGCCTGAAGGTGC; MyoD: F, CGACTGCCTGTCCAGCATAG, R, GGACACTGAGGGGTGGAGTC.

### 4.4. Histopathology and Immunohistochemistry of Rabbit Tissues

Five larynges were harvested from each group for histological analysis following euthanasia one month after nerve transection. Specimens were embedded in paraffin blocks and 4-um-thick coronal sections were made using a microtome. The sections were stained with H&E using standard pathology department protocols.

For immunohistochemical examination, the sections were incubated overnight at 4 °C with a 1:200 dilution of anti-fast myosin skeletal heavy-chain antibody (Abcam, Cambridge, UK) or anti-Met (c-Met) antibody (Abcam) in 1% (*v*/*v*) normal goat serum, and 1% (*w*/*v*) bis(trimethylsilyl)acetamide (BSA) in PBS. After washing with PBS, the sections were incubated with a 1:500 dilution of Alexa fluor 488-conjugated anti-mouse IgG antibodies (Cell Signaling Technology, Danvers, MA, USA) or Alexa Fluor 594-conjugated anti-mouse IgG antibodies (1:500 dilution; Cell Signaling Technology) for 1 h in the dark at room temperature. The sections were then counterstained with hematoxylin, rehydrated in a series of graded alcohol/water mixtures and xylene, and covered with coverslips. The stained samples were observed using an inverted light microscope (Eclipse TE300; Nikon) equipped with a digital camera. Stained areas were measured using ImageJ software (National Institutes of Health, Bethesda, MD, USA). Immunohistochemical staining was evaluated by calculating the stained pixel area using ImageJ in a blinded fashion.

### 4.5. Statistical Analysis

All experiments were repeated at least three times. Data are expressed as the mean ± standard error of the mean (SEM). All statistical tests were performed using GraphPad Prism 5.0 software (GraphPad Software Inc., San Diego, CA, USA). One-way analysis of variance followed by post-hoc Tukey’s tests were used to compare multiple groups. *p*-values <0.05 were considered to indicate statistical significance.

## Data Availability

The datasets generated during and/or analyzed during the current study are available from the corresponding author on reasonable request.

## References

[1] Onwordi L.N., Al Yaghchi C. (2021). Airway Glottic Insufficiency. StatPearls.

[2] Angerstein W. (2018). Vocal Changes and Laryngeal Modifications in the Elderly (Presbyphonia and Presbylarynx). Laryngorhinootologie.

[3] Santos M., Freitas S.V., Dias D., Costa J., Coutinho M., Sousa C.A., da Silva Á M. (2021). Presbylarynx: Does Body Muscle Mass Correlate with Vocal Atrophy? A Prospective Case Control Study. Laryngoscope.

[4] Takano S., Kimura M., Nito T., Imagawa H., Sakakibara K., Tayama N. (2010). Clinical analysis of presbylarynx--vocal fold atrophy in elderly individuals. Auris Nasus Larynx.

[5] Araki K., Suzuki H., Uno K., Tomifuji M., Shiotani A. (2018). Gene Therapy for Recurrent Laryngeal Nerve Injury. Genes.

[6] Makiyama K., Hirano S. (2017). Aging Voice.

[7] Müller A.H. (2020). Laryngeal Synkinesis: A Viable Condition for Laryngeal Pacing. Adv. Otorhinolaryngol..

[8] Wang W., Chen D., Chen S., Li D., Li M., Xia S., Zheng H. (2011). Laryngeal reinnervation using ansa cervicalis for thyroid surgery-related unilateral vocal fold paralysis: A long-term outcome analysis of 237 cases. PLoS ONE.

[9] van Lith-Bijl J.T., Desuter G.R.R. (2020). Laryngeal Reinnervation: The History and Where We Stand Now. Adv. Otorhinolaryngol..

[10] Hirano S., Kawamoto A., Tateya I., Mizuta M., Kishimoto Y., Hiwatashi N., Kawai Y., Tsuji T., Suzuki R., Kaneko M. (2018). A phase I/II exploratory clinical trial for intracordal injection of recombinant hepatocyte growth factor for vocal fold scar and sulcus. J. Tissue Eng. Regen. Med..

[11] Sheehan S.M., Tatsumi R., Temm-Grove C.J., Allen R.E. (2000). HGF is an autocrine growth factor for skeletal muscle satellite cells in vitro. Muscle Nerve.

[12] Syverud B.C., VanDusen K.W., Larkin L.M. (2016). Growth Factors for Skeletal Muscle Tissue Engineering. Cells Tissues Organs.

[13] Hiwatashi N., Hirano S., Mizuta M., Kobayashi T., Kawai Y., Kanemaru S.I., Nakamura T., Ito J., Kawai K., Suzuki S. (2017). The efficacy of a novel collagen-gelatin scaffold with basic fibroblast growth factor for the treatment of vocal fold scar. J. Tissue Eng. Regen. Med..

[14] Choi J.S., Lee S., Kim D.Y., Kim Y.M., Kim M.S., Lim J.Y. (2015). Functional remodeling after vocal fold injury by small intestinal submucosa gel containing hepatocyte growth factor. Biomaterials.

[15] Choi Y.H., Ahn H.J., Park M.R., Han M.J., Lee J.H., Kwon S.K. (2019). Dual growth factor-immobilized bioactive injection material for enhanced treatment of glottal insufficiency. Acta Biomater..

[16] Kim Y.C., Lee J., An J.N., Kim J.H., Choi Y.W., Li L., Kwon S.H., Lee M.Y., Lee B., Jeong J.G. (2019). Renoprotective effects of a novel cMet agonistic antibody on kidney fibrosis. Sci. Rep..

[17] Dhawan J., Rando T.A. (2005). Stem cells in postnatal myogenesis: Molecular mechanisms of satellite cell quiescence, activation and replenishment. Trends Cell Biol..

[18] Adreani C.M., Li Z.B., Lehar M., Southwood L.L., Habecker P.L., Flint P.W., Parente E.J. (2006). Myosin heavy chain composition in normal and atrophic equine laryngeal muscle. Vet. Pathol..

[19] Shiotani A., Flint P.W. (1998). Myosin heavy chain composition in rat laryngeal muscles after denervation. Laryngoscope.

[20] Flint P.W., Nakagawa H., Shiotani A., Coleman M.E., O’Malley B.W. (2004). Effects of insulin-like growth factor-1 gene transfer on myosin heavy chains in denervated rat laryngeal muscle. Laryngoscope.

[21] Tsukamoto S., Shibasaki A., Naka A., Saito H., Iida K. (2018). Lactate Promotes Myoblast Differentiation and Myotube Hypertrophy via a Pathway Involving MyoD In Vitro and Enhances Muscle Regeneration In Vivo. Int. J. Mol. Sci..

